# Vascular Endothelial Growth Factor-Related Pathways in Hemato-Lymphoid Malignancies

**DOI:** 10.1155/2010/729725

**Published:** 2010-05-24

**Authors:** Michael Medinger, Natalie Fischer, Alexandar Tzankov

**Affiliations:** ^1^Department of Hematology, University Hospital Basel, Petersgraben 4, 4031 Basel, Switzerland; ^2^Clinic for Medical Oncology, University Hospital Basel, Petersgraben 4, 4031 Basel, Switzerland; ^3^Institute of Pathology, University Hospital Basel, Schoenbeinstrasse 40, 4031 Basel, Switzerland

## Abstract

Angiogenesis is essential for malignant tumor growth. This has been documented for solid tumors, and there is an emerging evidence suggesting that tumor progression of hematolymphoid malignancies also depends on the induction of new blood vessel formation. The most important proangiogenic agent is vascular endothelial growth factor (VEGF), activating VEGF receptors 1 and 2. The available data on angiogenesis in hemato-lymphoid malignancies, such as acute leukemias, myelodysplastic syndromes, myeloproliferative neoplasms, multiple myeloma, and lymphomas, point towards the significance of autocrine and paracrine VEGF-mediated effects for proliferation and survival of leukemia/lymphoma cells in addition to tumor vascularization. Antiangiogenic strategies have become an important therapeutic modality for solid tumors. Several antiangiogenic agents targeting VEGF-related pathways are also being utilized in clinical trials for the treatment of hemato-lymphoid malignancies, and in some instances these pathways have emerged as promising therapeutic targets. This review summarizes recent advances in the basic understanding of the role of angiogenesis in hemato-lymphoid malignancies and the translation of such basic findings into clinical studies.

## 1. Introduction

New blood vessel formation (angiogenesis) is fundamental to tumor growth and spread. In adults, physiological angiogenesis is limited to a small number of specific processes, such as wound healing, tissue repair, and the female reproductive cycle [1]. The pioneering work of Judah Folkman led to the recognition that angiogenesis plays an important role in tumor development, progression, and metastasis [2]. Tumors require nutrients and oxygen to grow, and new blood vessels, formed by angiogenesis, provide these substrates. Tumor blood vessels are generated by various mechanisms, such as cooption of the existing vascular network, expansion of the host vascular network by budding of endothelial sprouts (sprouting angiogenesis), remodeling and expansion of vessels by the insertion of interstitial tissue columns into the lumen of preexisting vessels (intussusceptive angiogenesis), and homing of endothelial cell precursors (EPC; CEP) from the bone marrow or peripheral blood into the endothelial lining of neovessels (vasculogenesis) [3]. Bone marrow-derived progenitor cells contribute significantly to neovascularization in a variety of tumors [4, 5]. 

**Figure 1:**
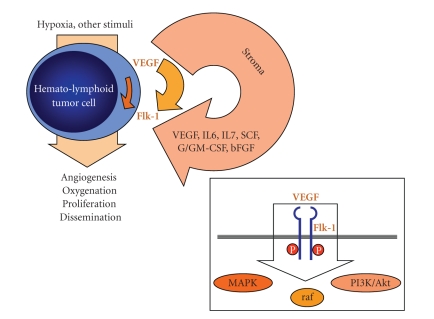
Possible vascular endothelial growth factor- (VEGF) and VEGF receptor-related (e.g., Flk-1, i.e., VEGFR-2) autocrine and paracrine loops in hemato-lymphoid neoplasms; insert: receptor tyrosine kinase activity and signaling cascades through VEGFR-2.

The key mediator of angiogenesis is the vascular endothelial growth factor (VEGF). Its expression is regulated by a plethora of intrinsic and extrinsic factors, with hypoxia and hypoglycemia being the major stimuli [6]. Hypoxia-induced transcription of *VEGF* mRNA is mediated by binding of hypoxia-inducible factor 1 (HIF-1) [7]. Cytokines may also modulate angiogenesis by regulating *VEGF* expression, for example, tumor necrosis factor (TNF)-*α* increases *VEGF* mRNA in glioma cells [8], and transforming growth factor (TGF)-*β* results in the induction of *VEGF* mRNA and protein in human lung adenocarcinoma cells [9]. In solid tumors, intratumoral hypoxia and HIF-1 mediation have been found to be a key angiogenesis triggering event [10]. Less is known about the exact trigger mechanisms of VEGF expression in hemato-lymphoid tumors, but mechanisms analogous to those observed in solid tumors are anticipated [11, 12]. Tight control of angiogenesis is maintained by a balance of endogenous antiangiogenic and proangiogenic factors. VEGF has a key, rate-limiting role in promoting tumor angiogenesis and exerts its effects by binding to one of three tyrosine kinase receptors (Figure 1): VEGF receptor-1 (VEGFR-1; fms-like tyrosine kinase-1, Flt-1), VEGFR-2 (human kinase domain region, KDR/murine fetal liver kinase-1, Flk-1), and VEGFR-3 (Flt-4). VEGFR-1 (ligands include VEGF-A, -B, and placental growth factor [PIGF]) and VEGFR-2 (ligands include VEGF-A, -C, and -D) are predominantly expressed on vascular endothelial cells, and activation of VEGFR-2 appears to be both necessary and sufficient to mediate VEGF-dependent angiogenesis and induction of vascular permeability [13, 14]. Both receptor tyrosine kinases are expressed in all adult endothelial cells except the brain. VEGFR-1 is also expressed on hematopoietic stem cells (HSC), vascular smooth muscle cells, monocytes, and leukemic cells [15, 16], while VEGFR-2 is expressed on endothelial progenitor cells and megakaryocytes [17, 18]. Although the exact contribution of VEGFR-1 signaling to angiogenesis is unclear, it has been shown that VEGFR-1 directly cooperates with VEGFR-2 via heterodimerization, as well as binding two additional VEGF homologues, VEGF-B and PIGF [19]. VEGFR-3, largely restricted to lymphatic endothelial cells, binds the VEGF homologues VEGF-C and VEGF-D and may play an important role in the regulation of lymphangiogenesis. 

VEGF and VEGFR represent significant anticancer therapy targets that elegantly bypass potential tumor-related treatment barriers [13]. VEGF signaling inhibition has been shown to result in significant tumor growth delay in a wide range of animal models [20]. In the case of VEGF, even a single *VEGF* allele knock-out led to embryonic lethality in mice [21]. The clinical benefit of this approach has also been confirmed, and concerted efforts in recent years have resulted in a number of novel antiangiogenic agents [22]. The first antiangiogenic agent to be approved was bevacizumab (Avastin, Genentech), a humanized anti-VEGF monoclonal antibody. Administration of bevacizumab, in combination with cytotoxic chemotherapy, conferred benefits to patients with metastatic colorectal cancer, nonsquamous, nonsmall cell lung cancer, and metastatic breast cancer [23–25], and it is now under investigation for patients with relapsed and refractory acute leukemia in combination with standard chemotherapy [26]. Additionally, two small-molecule inhibitors targeting VEGFR and other kinases, sorafenib (Nexavar, Bayer and Onyx pharmaceuticals) and sunitinib (Sutent, Pfizer), have been approved based on their efficacy in treating renal cell- and hepatocellular carcinoma [27, 28]. A growing number of antiangiogenics are now either in various stages of clinical development or in clinical use as components of standard regimens (Table 1). The major classes of antiangiogenic therapy include (1) direct anti-VEGF acting molecules (anti-VEGF antibodies, *VEGF*-antisense nucleotides), (2) immunomodulatory drugs (IMIDs) with antiangiogenic properties, (3) receptor tyrosine kinase inhibitors that target VEGFR signaling as well as receptors of other (proangiogenic) factors, (4) the antiendothelial approach of metronomic therapy, and (5) other new compounds targeting signaling downstream to proangiogenic growth factors, such as mammalian target of rapamycin (mTOR) inhibitors, histone deacetylases' (HDAC) inhibitors, and proteasome inhibitors. 

Several studies suggested that angiogenesis plays an important role, as might autocrine and paracrine VEGF/VEGFR-related loops in hemato-lymphoid malignancies such as acute and chronic leukemias, myelodysplastic syndromes (MDS), myeloproliferative neoplasms (MPN), lymphomas, and multiple myeloma (MM) [29–39] (Figure 1). Moreover, angiogenesis appears to be targeted even by conventional chemotherapy in hemato-lymphoid malignancies; for example, patients with acute myelogenous leukemia (AML) show increased microvessel density (MVD) in the bone marrow with subsequent MVD reduction under chemotherapy and return to normal levels in cases of complete remission (CR) [31]. This review will focus on the current knowledge of angiogenesis and antiangiogenic therapies (related to classes 1 to 3 of antiangiogenic treatment approaches) in hemato-lymphoid malignancies.

## 2. Acute Leukemias

**Figure 2:**
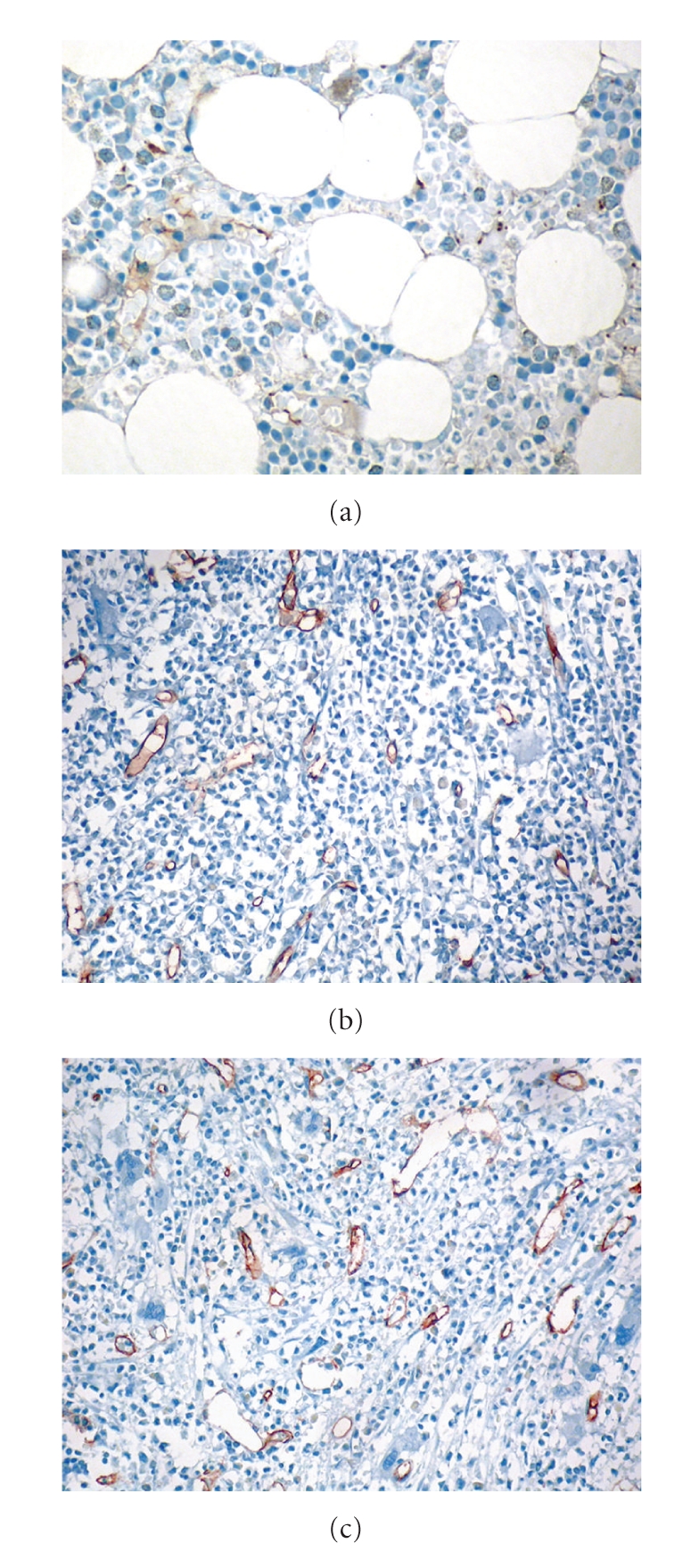
(a) Microvascular architecture in normal bone marrow highlighted by CD34; note single capillaries. (b) Increased microvessel density in acute myelogenous leukemia and in (c) myeloproliferative neoplasm; note dilated sinus and atypical megakaryocytes in the latter.

Leukemias have been ever since associated with angiogenesis since the AML cell line HL-60 was first used to clone the *VEGF *gene [40]. The first demonstration that leukemia progression might be accompanied by an increase of bone marrow vascularization was provided by Judah Folkman's group [41], who demonstrated that the bone marrow of acute lymphoblastic leukemia (ALL) patients had increased blood vessel content compared to normal counterparts. Detailed analysis of bone marrow sections from ALL patients led to the development of a model to illustrate their irregular, albeit abundant, bone marrow vasculature. Moreover, it was also shown that urine and peripheral blood samples from ALL patients contained elevated levels of proangiogenic growth factors, namely, basic fibroblast growth factor (bFGF) and VEGF, which correlated with the increase of bone marrow angiogenesis [41, 42]. These studies raised the question of whether the growth of other types of hemato-lymphoid malignancies is also accompanied by increased angiogenesis, while proving that the basic molecular/cellular mechanisms occurring during leukemia expansion might be similar to those seen in solid tumors. The existence of an “angiogenesis switch”, first proposed for solid tumors [43], was therefore suggested to apply to hemato-lymphoid malignancies as well. “Angiogenesis switch” in leukemia is documented by increased bone marrow MVD (Figures 2(a) and 2(b)), increased expression of HIF-1, multiple proangiogenic factors (VEGF, bFGF, angiopoietin-2), soluble VEGFR, and decreased expression of endogenous angiogenesis inhibitors, such as thrombospondin-1 [11, 12]. 

In a recent study by Norén-Nyström et al. [44], MVD, analyzed on 185 bone marrow biopsies, was higher in T-ALL compared to B-ALL. In the B-ALL group, cases with t(12;21) were characterized by a low MVD, while patients with hyperdiploid leukemia showed a high MVD. There was a correlation between MVD and white blood cell count in high-risk B-ALL patients. In addition, patients with a high marrow reticulin fiber density and high MVD had an unfavorable outcome. Similarly, in previously untreated AML, increased levels of plasma VEGF correlated with reduced survival and lower remission rates [45]. Moreover, the level of plasma/serum VEGF correlated with the number of circulating blasts [46], indicating the probable cellular origin of this proangiogenic factor. Such in vivo clinical studies are further supported by in vitro demonstrations of the capacity of leukemia cells to produce proangiogenic growth factors such as VEGF and bFGF [47–49]. Importantly, leukemia cells release increased amounts of proangiogenic factors in response to proinflammatory molecules, suggesting interactions with other cell types (e.g., the bone marrow stroma). In contrast to the abundant literature demonstrating that acute leukemia cells secrete significant amounts of angiogenesis activators such as VEGF, fewer studies have addressed the possibility that reduced production of angiogenesis inhibitors by these cells might also trigger the neovascularization process by shifting the local (bone marrow) angiogenesis balance [50, 51]. In addition to the modulation of bone marrow angiogenesis by leukemia cells, it was demonstrated that subsets of cases express endothelial-specific tyrosine kinase receptors, such as VEGFR-1, -2, and -3, or members of the FGF receptor family [49, 52, 53]. Recent studies showed that even hematopoietic stem cells (HSC) express VEGFR and are capable of generating functional autocrine loops that support their proliferation and survival [54], raising the question of whether this might be of relevance for leukemic stem cells. 

### 2.1. Antiangiogenic Therapy in Acute Leukemias

Bevacizumab is a humanized murine antihuman VEGF monoclonal IgG_1_ antibody that blocks the binding of human VEGF to its receptors, thereby also disrupting autocrine and paracrine survival mechanisms mediated by VEGFR-1 and VEGFR-2 [55]. In a Phase II clinical trial by Karp et al., bevacizumab was administered after chemotherapy to adults with refractory or relapsed AML [26]. Bevacizumab 10 mg/kg was administered on day 8 after cytarabine beginning day 1 and mitoxantrone beginning day 4. Forty-eight adults received induction therapy and the overall response was 23 of 48 (48%), with CR in 16 (33%). Eighteen patients (14 CR and 4 partial responses) underwent one consolidation cycle and 5 (3 CR and 2 partial responses) underwent allogeneic transplant. Median overall and disease-free survivals for CR patients were 16.2 months (64%, 1 year) and 7 months (35%, 1 year), respectively. Bone marrow samples demonstrated marked MVD decrease after bevacizumab administration. VEGF was detected in pretreatment serum in 67% of patients tested, increased by day 8 to 52%, and decreased to 93% (67% undetectable) 2 hours after bevacizumab administration. Currently, bevacizumab is being evaluated as treatment option for newly diagnosed AML in combination with cytarabine and idarubicin in a phase II study.

Thalidomide, originally marketed as a sedative and antiemetic drug, was withdrawn from use subsequent to reports of teratogenicity in the 1960s. The initial use of thalidomide was the treatment of erythema nodosum leprosum. It has been shown that thalidomide has important immunomodulatory effects in that it decreases TNF-*α* synthesis and selectively modulates T-cell subsets, shifting the T-cell population towards T helpers [56]. The interest in thalidomide as an antineoplastic agent rose after demonstration of its antiangiogenic activity in a rabbit model of corneal neovascularization that was induced in response to bFGF [57]. This report led to thalidomide application in MM, where it demonstrated a clinical benefit. Thalidomide and the newer IMIDs (e.g., lenalidomide) have been shown to significantly decrease the expression of the proangiogenic factors VEGF and interleukin-6 (IL-6) in MM [58]. The newer IMIDs were found to have 2-3 times more potent antiangiogenic activity than thalidomide in various in vivo assays [59]. The antiangiogenic activity of IMIDs has been shown to be independent of their immunomodulatory effects [60]. In AML patients, thalidomide therapy was assessed alone and in combination with other compounds. In a phase II study by Thomas et al. [61], thalidomide was analyzed in patients with relapsed or refractory AML previously treated with cytarabine-containing regimens. A total of 16 patients were treated with 200–800 mg/d oral thalidomide. Overall, one patient (6%) achieved CR lasting for 36 months, and two patients had a transient reduction in marrow blasts from 8% and 7% to less than 5%. There was no correlation between reduction of angiogenesis marker levels and response. In a phase I/II trial by Steins et al. [62], a dose-escalating trial was performed to study the safety and efficacy of thalidomide in 20 AML patients. Thirteen patients were assessable for both toxicity and response, tolerating a maximum dose of 200–400 mg/d for at least 1 month. Overall, adverse events were fatigue, constipation, rash, and neuropathy (grades 1 to 2 in most patients). In 4 patients, a partial response, defined as reduction of at least 50% of the blast cell infiltration in the bone marrow accompanied by increases of platelet counts and hemoglobin values, was observed. In parallel, MVD significantly decreased in these 5 patients during treatment with thalidomide. In a study by Barr et al. [63], thalidomide was examined in combination with fludarabine, carboplatin, and topotecan in 42 patients with poor prognosis AML, and 10 of 42 (24%) patients achieved a CR. Serious thrombotic adverse events were observed in 5 patients, suggesting that the combination of cytotoxic chemotherapy and thalidomide may be thrombogenic despite significant thrombocytopenia. VEGF levels did not correlate with response to therapy, while a trend towards decreased MVD was noted in patients who achieved CR.

Small tyrosine kinase inhibitors that target VEGFR are a further important class of antiangiogenic drugs with application to AML, although their efficacy in hemato-lymphoid neoplasias, especially AML, might be attributable to inhibition of a variety of pathways, particularly those related to c-kit and flt3.

Vatalanib (formerly PTK787/ZK 222584) is an oral angiogenesis inhibitor that is active against VEGFR and PDGFR tyrosine kinases, thereby offering a novel approach to inhibiting tumor growth [64] by interfering with the ATP binding sites of VEGFR. In our phase I study, vatalanib was well tolerated and showed clinical activity in a variety of solid tumors [65]. In MM, its action primarily reduces the number of tumor microvessels and dilates the remaining vessels [66, 67]. Ongoing studies are now evaluating the efficacy of vatalanib in combination with imatinib in a phase I/II trial for patients with AML, PMF, and blast phase of chronic myelogenous leukemia. Vatalanib was studied in a phase I clinical trial alone or in combination with cytosine-arabinoside and daunorubicin in patients with MDS and AML [68]. Sixty-three patients received vatalanib at doses of 500–1000 mg/bid orally. At 1000 mg/bid, dose-limiting toxicities resulting in lethargy, hypertension, nausea, emesis, and anorexia were observed. CR was observed in 5 of 17 evaluable AML patients treated with vatalanib combined with chemotherapy. The authors concluded that vatalanib is generally well tolerated and can be given in combination with chemotherapy in patients with MDS and AML. In a recent study by Barbarroja et al. [69], vatalanib was examined in combination with idarubicin in 4 AML cell lines and 7 AML patient samples. Vatalanib decreased VEGF levels and VEGFR phosphorylation in AML cells, which showed *FLT3* internal tandem reduplications/mutations (ITD), raising questions about the actual targeted tyrosine kinase (VEGFR of flt3). 

Cediranib (AZD2171, Recentin) is a potent inhibitor of both VEGFR-1 and VEGFR-2; it also has activity against c-kit, PDGFR-*β*, and VEGFR-3 at nanomolar concentrations [70]. In our study, cediranib was well tolerated up to 45 mg/d in patients with a broad range of solid tumors [71], with the most common adverse side-effects being diarrhea, dysphonia, and hypertension. In a phase I study with cediranib in 35 AML patients, the most common adverse events were diarrhea, hypertension, and fatigue. Six patients experienced an objective response (3 each at 20 and 30 mg). Dose- and time-dependent reductions of soluble VEGFR-2 were observed, and there was a correlation between cediranib exposure and plasma VEGF levels [72].

## 3. Myelodysplastic Syndromes

In MDS, VEGF is overexpressed by immature myeloid cells in the bone marrow and associated with increased bone marrow vascularity [52]. MVD parallels disease progression from refractory anemia to secondary AML [32]. MDS patients also have increased proangiogenic factors in peripheral blood [73]. Higher levels of VEGF were found by immunohistochemistry and corroborated by reverse transcriptase-polymerase chain reaction in patients with refractory anemia with excess blasts (RAEB) and RAEB in transformation (RAEB-T), compared to patients with refractory anemia (RA) and with ringed sideroblasts (RARS) or normal bone marrow controls [74]. These differences were thought to result from expression of VEGF in immature myeloid cells in RAEB and RAEB-T. To evaluate the interplay between angiogenesis and cytokines, we conducted a study of 89 MDS cases and showed [34] that TNF-*α* expression and bone marrow MVD correlated with each other as well. Importantly, thalidomide, a drug that modulates T-cell function and inhibits TNF-*α* activity as well as angiogenesis, is under investigation in clinical trials for the treatment of MDS [29, 75]. 

### 3.1. Antiangiogenic Therapy in Myelodysplastic Syndromes

A combination therapy of thalidomide and 5-azacytidine, a hypomethylating drug, was assessed in 40 patients with MDS and AML [76]. Hematological improvement was observed in 15 of 36 patients (42%), stable disease was observed in 5 of 36 patients (14%), 10 of 36 patients (28%) had disease progression, and six had CR. Lenalidomide, a synthetic compound derived by modifying the chemical structure of thalidomide, has also shown immunomodulatory and antiangiogenic properties and lower adverse effects rates [77]. Lenalidomide was investigated in a study of 148 MDS patients with 5q deletion [78]; 112 patients (76%) had a reduced need for transfusions, and 99 (67%) eliminated the need entirely regardless of karyotype complexity. Among 85 evaluable patients, 62 showed cytogenetic improvement, and 38 of that 62 showed complete cytogenetic remission. Therefore, lenalidomide was approved as a monotherapy for the treatment of transfusion-dependent MDS patients with 5q deletion with or without additional cytogenetic abnormalities.

## 4. Multiple Myeloma

MM is characterized by proliferation of malignant plasma cells that accumulate in the bone marrow and often produce a monoclonal immunoglobulin. MM was the first hemato-lymphoid malignancy in which increased angiogenesis was detected [36, 79]. New vessel formation in the bone marrow seems to play an important role in the pathogenesis of MM [80, 81]. Increased bone marrow MVD in patients with MM also appears to be an important prognostic factor [82]. Malignant plasma cells can secrete various cytokines, including VEGF, bFGF, and hepatocyte growth factor (HGF), all known for their proangiogenic activity [83]. It has been shown that MM cells are capable of secreting VEGF in response to IL-6 stimulation, and in response to this VEGF stimulation, microvascular endothelial cells and bone marrow stromal cells in turn secrete IL-6, a potent growth factor for malignant plasma cells, thus closing a paracrine loop [84, 85]. Rajkumar et al. showed a gradual increase of bone marrow angiogenesis along the disease spectrum from monoclonal gammopathy of undetermined significance (MGUS) to smoldering MM, newly diagnosed MM, and relapsed MM [86], though the expression levels of VEGF, bFGF, and their receptors were similar among MGUS, smoldering MM, and newly diagnosed MM [87], suggesting that MVD increase in plasma cell neoplasias could be a function of chronology. In a recent study, Pour et al. examined 96 MM patients at diagnosis and after high-dose chemotherapy with regard to angiogenesis factor/inhibitor concentrations in the peripheral blood and bone marrow plasma [88]. Based on a significant decrease of VEGF and hepatocyte growth factor levels, and a significant increase in TSP-1 thrombospondin-1 concentrations in the bone marrow plasma of patients who achieved complete or very good partial response versus those who had partial or no response, they concluded that a reduction in the rate of angiogenesis had occurred.

### 4.1. Antiangiogenic Therapy in Multiple Myeloma

Thalidomide monotherapy in a phase II trial of 84 patients with relapsed and refractory MM who had received doses ranging from 200 to 800 mg/d resulted in an overall response rate of 32%. The 2-year event-free and overall survival were 20 and 48%, respectively [89, 90]. In combination with dexamethasone, the response rate was 63% compared to 41% with dexamethasone alone in patients with newly diagnosed MM [91]. Subsequent to these studies, thalidomide was approved for the treatment of newly diagnosed MM. In 2 phase III trials, lenalidomide in combination with dexamethasone resulted in remarkable response rates and significantly less toxicity than thalidomide [92, 93], and increased the response rate from 22.5% to 59.2% compared to dexamethasone alone in patients with previously-treated relapsed/refractory MM. Lenalidomide was approved in combination with dexamethasone for second-line treatment of MM.

## 5. Myeloproliferative Neoplasms

The available data on angiogenesis and expression of VEGF and VEGFR in the bone marrow of patients with *BCR-ABL1-*negative MPN suggest a significant increase of MVD (Figure 2(c)), especially in primary myelofibrosis (PMF), which might inversely correlate with survival [30, 33, 94–97]. The identification of an acquired somatic mutation in the *JAK2* gene, resulting in a valine to phenylalanine substitution at position 617 (*JAK2-V617F*), has provided new insights into the pathogenesis of *BCR-ABL1-*negative MPN, which is found in most patients with polycythemia vera (PV) and in about 50% of patients with essential thrombocythemia (ET) and PMF [98, 99]. The correlations between angiogenesis and *JAK2* status in MPN have been addressed in two studies with contradictory results [100, 101]. In a recent study [30], we found a significantly increased MVD and VEGF expression in MPN compared to controls, particularly in cases with high *JAK2-V617F* mutant allele burdens. Our results imply that higher activity of Jak2-related pathways, as observed in cases with higher *JAK2-V617F* mutant allele burdens, may influence angiogenesis in MPN. This assumption is further supported by our observation that the number of VEGF expressing cells did not rise concurrent with the increasing *JAK2-V617F* mutant allele burden regardless of rising MVD. Further support is provided by the study of Zhu et al. showing that H-2g, a glucose analog of the blood group H antigen, mediates endothelial cell chemotaxis and induces expression of the proangiogenic factors VEGF and bFGF through pathways involving Jak2 and phosphoinositide-3 kinase that could be abolished by treatment with the respective inhibitors AG490 and LY294002 [102]. The importance of VEGF/Jak2/STAT5 pathways in angiogenesis is substantiated by evidence from another study as well suggesting tight interactions between VEGF and Jak2 [103]. Thus, it could be speculated that in at least some hemato-lymphoid neoplasms, such as *BCR-ABL1-*negative MPN, key tumor-related gate-keeping genetic mechanisms might also directly influence angiogenesis. A very recent study identified the *JAK2-V617F *mutation in microdissected endothelial cells form the liver veins of Budd-Chiari syndrome patients [104], raising the hypothesis that endothelial cells in PV are direct players in the neoplastic process.

### 5.1. Antiangiogenic Therapy in Myeloproliferative Neoplasms

In a phase II study of 44 PMF patients, the efficacy of thalidomide monotherapy was assessed [105]. Seventeen of 41 evaluable patients (41%) receiving treatment for at least 15 days showed a response. CR (without reversal of bone marrow fibrosis) was achieved in 4 patients (10%), partial response in 4 patients (10%), and hematological improvements of anemia, thrombopenia, and/or splenomegaly were observed in 9 patients (21%). A further phase II thalidomide and placebo study assessed the efficacy of thalidomide in the treatment of anemia in PMF [106]. The primary outcome was a 2 g/l increase of hemoglobin levels resulting in a 20% reduction of transfusion needs. At 180 days, in an intention-to-treat analysis, no difference was observed between the thalidomide and placebo groups with regard to hemoglobin levels. In phase II studies with lenalidomide monotherapy in patients with symptomatic PMF, the overall response rates were 22% for anemia, 33% for splenomegaly, and 50% for thrombocytopenia [107]. In a combination study of lenalidomide with prednisone in 40 PMF patients [108], responses were recorded in 12 patients (30%) and are ongoing in 10 (25%), with a median time to response of 12 weeks. Three patients (7.5%) had partial response and nine (22.5%) had clinical improvement lasting a median of 18 months. Overall response rates were 30% for anemia and 42% for splenomegaly. Interestingly, all eight *JAK2-V617F*-positive responders also experienced a reduction of the baseline mutant allele burden.

In another study, vatalanib was administered to 29 PMF patients at doses of 500 or 750 mg/bid. One patient (3%) achieved CR and 5 (17%) achieved clinical improvement. Cumulatively, these studies indicated that vatalanib had modest activity in PMF patients [109].

## 6. Lymphomas

**Figure 3:**
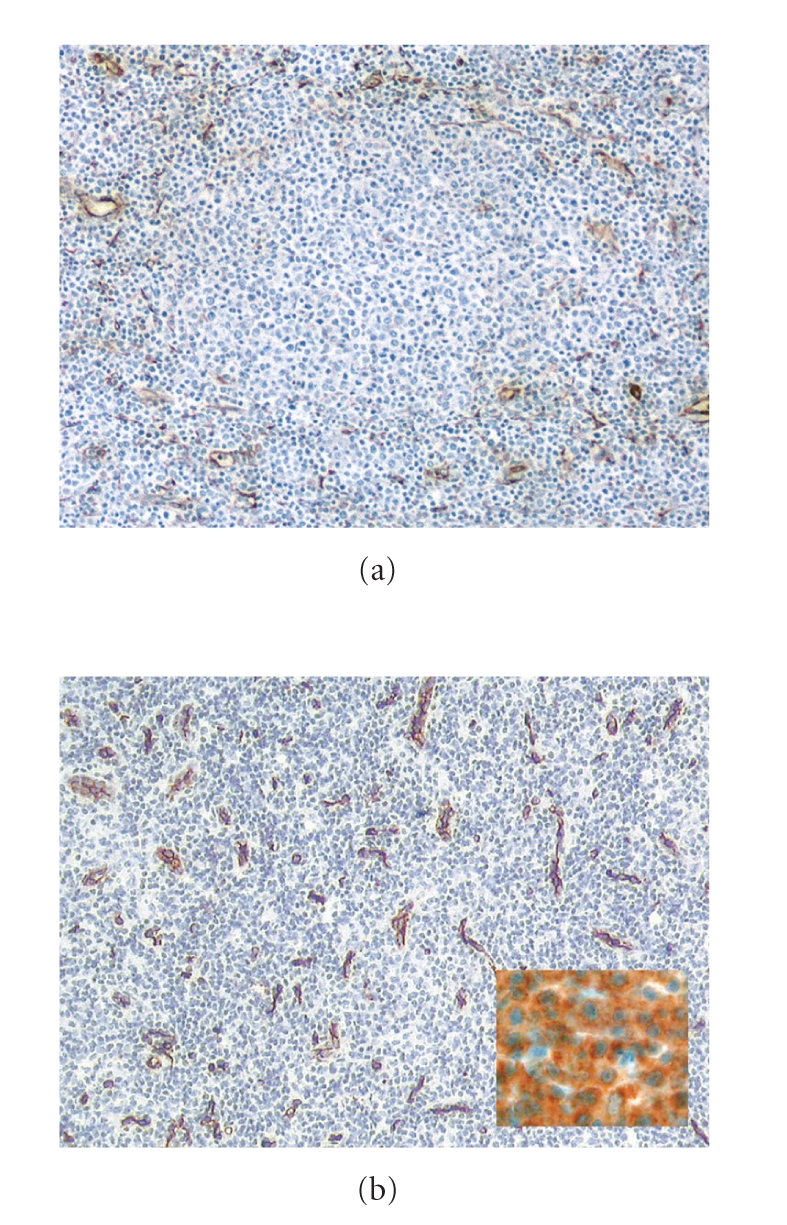
(a) Microvascular organization of the cortical (B-cell-) zone of a normal lymph node highlighted by CD34; note a perifollicular condensation of microvessels and paucity inside the follicle. (b) Increased microvessel density in a mantle cell lymphoma case; insert: vascular endothelial growth factor expression in a diffuse large B-cell lymphoma case highlighted by immunohistochemistry.

MVD is significantly higher in nodal lymphomas, particularly in those that are highly proliferative, than in reactive lymph nodes (Figures 3(a) and 3(b)) [35, 110–113]. In extranodal lymphomas, for example, cutaneous T-cell and B-cell lymphomas, MVD are higher than in normal skin or benign cutaneous lymphoproliferative disorders [114–116]. The interplay between lymphoma cells and tumor vessels is complex [117]; lymphoma-specific chromosomal aberrations such as t(8;14), t(11;14), and t(14;18) were discovered in tumor endothelial cells, calling into question the histogenesis of B-cell lymphoma vasculature [118]. Lymphoma growth and progression appear to be promoted by at least two distinct angiogenic mechanisms: autocrine stimulation of tumor cells via expression of VEGF and VEGFR by lymphoma cells, and paracrine influences of the proangiogenic tumor microenvironment on both local neovascular transformation and recruitment of circulating bone marrow-derived EPC [119]. The lymphoma microenvironment has been increasingly recognized as influencing neoplastic progression, in part by modulating angiogenic responses to distinct proangiogenic growth factors and cytokine milieu. In follicular lymphoma and diffuse large B-cell lymphoma (DLBCL), large-scale gene expression profiling studies demonstrated that genetic signatures expressed by stromal and infiltrating immune cells defined distinct prognostic groups [120, 121]. In the study of Lenz et al. [121], DLBCL gene-expression signatures correlated with survival. A multivariate model, created from three gene-expression signatures termed “germinal-center B-cell”, “stromal-1”, and “stromal-2”, predicted survival and was influenced by differences in immune cells, fibrosis, and particularly angiogenesis in the tumor microenvironment.

The prognostic and predictive value (as possible treatment target) of MVD and angiogenic factors in lymphomas is still controversial due to the heterogeneity of diseases, different classifications, and methods for analysis (immunohistochemistry, serum levels of angiogenic markers, mRNA extraction, etc.). In B-cell lymphomas, VEGF protein and mRNA have been identified in DLBCL, mantle cell lymphoma (MCL), central nervous system DLBCL, and viral-related lymphomas [122]. A large study of 200 patients showed that high pretreatment levels of both serum VEGF and bFGF were independent prognostic factors for survival in multivariate analysis [123]. In a study with *de novo* DLBCL treated with anthracycline-based therapy, increased tumor vascularity was associated with poor overall survival independent of the international prognostic index [124]. Our results on the in situ expression of VEGF in B-cell lymphomas did not suggest a prognostic value, but showed distinct expression patterns among the different entities with higher prevalence in DLBCL [35]. Since VEGF expression in lymphomas parallels their proliferative activity [110–112], our findings of strong VEGF expression (20 out of 109 cases, 18%) and highest MVD in DLBCL were not surprising (Figure 2(b)) [35]. We found no direct correlation between increased MVD and VEGF expression in DLBCL. 

Regarding MVD, follicular lymphoma is of special interest. Several studies have recognized increased vascularization in the perifollicular compartment of affected lymph nodes [35, 111, 113, 125]. The MVD inside the neoplastic follicles seems similar to that in reactive lymph nodes and somewhat lower than in DLBCL and MCL. Koster and Raemaekers [113] showed that in follicular lymphoma, increased vascularization is associated with improved clinical outcome. Furthermore, VEGF expression seemed not to be involved in follicular lymphoma angiogenesis.

In T-cell lymphomas, the *VEGF* gene is overexpressed in both microdissected lymphoma- and endothelial cells of angioimmunoblastic T-cell lymphoma (AITL) [126]. Accordingly, VEGF protein expression was also found in both types of cells in lymph nodes and bone marrow samples with AITL involvement. 

In Hodgkin lymphoma (HL), VEGF is expressed along with HIF-1, VEGFR-2, and platelet-derived endothelial growth factor (PDGF) and its receptor (PDGFR*α*) at both the protein and RNA levels [127, 128]. MVD seems also to be increased in HL, especially in progressive disease [128]. 

### 6.1. Antiangiogenic Therapy in Lymphomas

Given the low proportion of DLBCL cases strongly expressing VEGF and the lack of correlation between VEGF and MVD in DLBCL, it could be anticipated that bevacizumab application in DLBCL would be not very successful, explaining the low observed response rates. In a phase II study of the Southwest oncology group, single agent bevacizumab was examined in 52 DLBCL or MCL patients in first or second relapses of aggressive lymphomas [129]. Patients were treated with bevacizumab at 10 mg/kg every 2 weeks, resulting in a six-month progression-free survival of 16% with a response rate of 2%, and median duration of response or stable disease of 5.2 months (range 3.5–72.7). Treatment was generally well tolerated, with grade 3 hypertension being the most significant adverse side effect in two patients. Clinical trials combining active chemotherapy regimens with VEGF-targeted agents are currently in progress. Thalidomide was evaluated in a study of 19 patients with recurrent/refractory lymphomas until disease progression or prohibitive toxicity was observed [130]. One patient (5%) with evidence of recurrent gastric mucosa-associated lymphoid tissue B-cell lymphoma achieved CR, and 3 patients (16%) achieved stable disease. There is more promising data with lenalidomide treatment of indolent lymphomas, including follicular lymphoma [131, 132], with reported response rates about 30% in pretreated patients, but the significance of lenalidomide effects other than modulation of angiogenesis, for example, immunomodulation, should be considered in this instance.

Given this strong VEGF production in AITL, it is not surprising that the results of anti-angiogenesis therapy in relapsed AITL are promising. There are published case reports [133, 134] with successful bevacizumab treatment in AITL leading to remissions lasting for several months. One patient received 4th line bevacizumab leading to CR lasting for 10 months; another received 5th line bevacizumab leading to CR with such excellent tolerability that an allogeneic transplant could be planned. Furthermore, there is additional small, but promising, body of evidence in favor of thalidomide [135–137] and lenalidomide [http://www.asco .org/ASCOv2/Meetings/Abstracts?&vmview=abst_detail_ view&confID=65&abstractID=30959]. In peripheral T-cell lymphoma, antiangiogenic substances are not yet integrated in primary, curative-intended therapies. In the relapsed, palliative setting, however, there are some promising data, especially with lenalidomide (25 mg/d during 3 weeks in a 4-week cycle) [138], including unpublished data on 24 T-cell lymphomas, including 7 patients with AITL [http://www.asco.org/ASCOv2/Meetings/Abstracts?&vmview=abst_detail_view&confID=65&abstractID=30959]. In these heavily pretreated patients, the response rate was 30%, and long-lasting remissions were also observed in the responding patients. 

After a successful tumor growth delay by bevacizumab administration in a xenograft HL model, Reiners et al. also showed some promising effects of combined gemcitabine/bevacizumab regimen in heavily pretreated HL patients with multiple relapses [139]. In a combination study, thalidomide was examined with vinblastine in patients with refractory HL [140]; of the 11 patients, 4 showed a partial response to treatment (response rate 36%) and two patients had stable disease.

## 7. Conclusion

Angiogenesis is essential to the development of hemato-lymphoid malignancies, including acute and chronic leukemias, MPN, and lymphomas. In such instances, VEGF/VEGFR-related pathways are the most relevant regulators of neoangiogenesis, vasculogenesis, and recruitment of endothelial progenitor cells. Furthermore, VEGF/VEGFR interactions can stimulate proliferation, migration, and survival of leukemia/lymphoma cells by autocrine and paracrine loops. Finally, in some hemato-lymphoid neoplasms, VEGF/VEGFR-related pathways represent a promising therapeutic target.

## Figures and Tables

**Table 1:** Summary of clinical trials and approved antiangiogenic therapies in hemato-lymphoid malignancies.

Drug class	Target	Study entities	Approved for
*Anti-VEGF strategies*			
Bevacizumab (Avastin)	VEGF-A	AML, MDS, CLL, CML, NHL, MM	Metastatic colorectal cancer, NSCLC, breast cancer

*RTK inhibitors*			
Vatalanib	VEGFR1-3, PDGFR*β*, CD117	AML, PMF, MDS, CML, DLBCL, MM	
Cediranib (Recentin)	VEGFR1-3, PDGFR*β*, CD117	AML, MDS, CLL	

*Immunomodulators*			
Thalidomide		AML, MDS, MPN, CLL, NHL, MM	MM
Lenalidomide (Revlimid)		AML, MDS, CLL, NHL	MM, 5q-MDS

## Abbreviations

AML: Acute myelogenous leukemia
DLBCL: Diffuse large B-cell lymphoma
CLL: Chronic lymphocytic leukemia
NHL: Non-Hodgkin lymphoma
NSCLC: Nonsmall cell lung cancer
MDS: Myelodysplastic syndrome
MM: Multiple myeloma
MPN: Myeloproliferative neoplasm
PDGFR: Platelet derived growth factor receptor
PMF: Primary myelofibrosis
RTK: Receptor tyrosine kinase
VEGF: Vascular endothelial growth factor (receptor).
